# Phenotypic effects of paralogous ribosomal proteins bL31A and bL31B in *E. coli*

**DOI:** 10.1038/s41598-020-68582-2

**Published:** 2020-07-15

**Authors:** Silva Lilleorg, Kaspar Reier, Pavel Volõnkin, Jaanus Remme, Aivar Liiv

**Affiliations:** Institute of Molecular and Cell Biology, University of Tartu, Riia street 23B, 51010 Tartu, Estonia

**Keywords:** Ribosomal proteins, Bacteria, Molecular biology, Translation, Ribosome

## Abstract

Ribosomes are essential macromolecular complexes conducting protein biosynthesis in all domains of life. Cells can have heterogeneous ribosomes, i.e. ribosomes with various ribosomal RNA and ribosomal protein (r-protein) composition. However, the functional importance of heterogeneous ribosomes has remained elusive. One of the possible sources for ribosome heterogeneity is provided by paralogous r-proteins. In *E. coli*, ribosomal protein bL31 has two paralogs: bL31A encoded by *rpmE* and bL31B encoded by *ykgM*. This study investigates phenotypic effects of these ribosomal protein paralogs using bacterial strains expressing only bL31A or bL31B. We show that bL31A confers higher fitness to *E. coli* under lower temperatures. In addition, bL31A and bL31B have different effects on translation reading frame maintenance and apparent translation processivity in vivo as demonstrated by dual luciferase assay. In general, this study demonstrates that ribosomal protein paralog composition (bL31A versus bL31B) can affect cell growth and translation outcome.

## Introduction

To survive, grow and reproduce organisms need proteins that function as enzymes, messengers, structural components of intra- and intercellular structures, transport and storage molecules. In addition to proper functionality, proteins have to be available in cells at the right time, place and in adequate amount to ensure/contribute to organism’s fitness/viability. Therefore, regulation of protein synthesis—translation—is of central importance in molecular biology. Translation is a highly complex process comprising an mRNA, aminoacylated tRNA-s, several protein factors and ribosomes and it can be regulated by various mechanisms^1, 2^.

An emerging paradigm is ribosome composition mediated translational control^3^ that is based on the hypothesis that ribosome can regulate translation via its composition. This concept is based on the accumulating evidence that eukaryotic and bacterial cells produce heterogeneous ribosomes, i.e. ribosomes containing alternative components^4, 5^. Ribosome heterogeneity has various sources: rRNA sequence and modifications, ribosomal protein (r-protein) stoichiometry, modifications and paralogs^6^. Therefore, the potential of creating ribosomes with various composition is enormous^7^.

An intriguing question is whether ribosome heterogeneity is functional. It means that first, biochemically different ribosomes are present in vivo under adverse growth conditions and second, that these variant ribosomes affect cell physiology via protein synthesis^8^. Recently, functional ribosome heterogeneity has been defined as “variations in ribosome composition that influence its activity, thereby changing the output of translation”^9^. As translation initiation is the main regulatory checkpoint for protein synthesis most research on the functionality of ribosome heterogeneity has been focused on this step of translation^6^. Specifically, one possible viewpoint to ribosome composition mediated translational control has been ribosome filter hypothesis stating that ribosomes may due to variations in their composition be able to preferentially bind different mRNA-s and enhance their translation^10^. On the other hand, recent literature points out that heterogeneous ribosomes may modulate various aspects of protein synthesis like fidelity and rate of elongation and other aspects of translation initiation beyond selectivity towards specific mRNAs^4, 9, 11^.

In *Bacteria* recent studies on rRNA sequence heterogeneity demonstrate that it directly contributes to stress adaptation^12, 13^. *Escherichia coli* and *Vibrio vulnificus* produce ribosomes with different rRNA sequences. After environmental conditions have changed these ribosomes were shown to translate stress response mRNA-s yielding in phenotypic effects like altered cell motility and biofilm formation in *E. coli*^12^ or better tolerance to heat shock and ability to use glycerol as the only carbon source in *V. vulnificus*^13^. In addition to rRNA sequence variations, bacterial ribosomes can contain differently modified rRNA^14^ and ribosomal proteins^15^ and substoichiometric amounts of r-proteins^16, 17^ but their functional importance has remained elusive.

Another possibility to create heterogeneous ribosomes is to produce ribosomes differing in their r-protein paralog content. In 2012 about half of the sequenced bacterial genomes encoded at least one paralogous r-protein^18^. Having paralogs is generally not a widespread feature of r-proteins: 46.5% of bacterial genomes have paralogous genes for one to three r-proteins. Interestingly, some r-proteins (seven) do not have any paralogs in the 995 completely sequenced bacterial genomes^18^ whereas others like bL33, bL31, bL36 and uS14 are duplicated in more than hundred bacterial genomes^18, 19^. Typically, paralogs of the latter group have fairly different amino acid sequences (identity < 50%)^18^.

Another characteristic feature of bacterial r-protein paralogs is the presence of a zinc-binding motif in one paralog^19^. The expression of different non-zinc binding paralogs is controlled by zinc-dependent Zur repressor and thus enhanced under zinc-limiting conditions^20–22^. In general, non-zinc binding paralogs are suggested to contribute to adaptation to zinc-limiting conditions^22, 23^. Based on the studies of bL31 it has been proposed that the zinc-binding paralog functions as a zinc storage protein in ribosomes that is replaced by the non-zinc binding paralog under zinc-deficient conditions thus liberating zinc ions for essential cellular functions^20, 24^. In addition, studies of uS14 have led to the idea that the non-zinc binding paralog is needed for 30S biogenesis under zinc-limiting conditions^25^. In both cases the functional role of r-protein paralogs in ribosomes was not addressed. In *Mycobacterium smegmatis*, contradictory results about the activity of ribosomes containing zinc-independent r-protein paralogs have been reported^26, 27^. Recently, ribosomes differing in their bS18 paralog content were shown to translate a gene set differently and to have defective translation initiation^27^. In addition, the entire operon encoding 4 non-zinc binding r-protein paralogs (bL28, bL33, uS14, bS18) has been demonstrated to be important for morphogenesis of *M. smegmatis* during zinc-limitation^22^.

In *E. coli*, two r-proteins bL31 and bL36 have each two paralogs^19^ referred to as bL31A, bL31B and bL36A and bL36B. In both cases, one of the paralogs (bL31A, bL36A, encoded by *rpmE* and *rpmJ* respectively) has a zinc-binding motif whereas the other (bL31B, bL36B encoded by *ykgM* and *ykgO* respectively) has not^19, 28^. *ykgM* and *ykgO* are located in the same operon^19^ regulated by transcription repressor Zur^29^ whereas *rpmE* and *rpmJ* are in separate operons^30^. All four proteins—bL31A (7.9 kDa), bL31B (9.9 kDa), bL36A (4.4 kDa) and bL36B (5.5 kDa)—have been identified in wild type *E. coli* ribosomes by quantitative MS^31^. Interestingly, in exponential growth phase bL31A and bL36A are the prevalent paralogs in ribosomes whereas in the stationary phase the majority of ribosomes contains bL31B and bL36B^31^. The switch between zinc- and non-zinc binding paralogs has been thought to be a mechanism to cope with zinc deficiency^24^. However, this may not be the only reason as enough zinc was present in these experiments and replacement of bL31A by bL31B was also found during stationary growth phase in the presence of zinc^31^.

It is noteworthy that although the amino acid sequence identity between bL31A and bL31B is low (< 40%) they occupy principally the same position in the ribosome^29^. bL31 is located in the central protuberance of the large subunit giving multiple interactions with the head of the small subunit^32, 33^. bL31B has an extra loop located between subunits and protruding outside the ribosome^31^. Based on its position in this highly dynamic region of the ribosome bL31 has been proposed^33^ and shown^34^ to contribute to translation fidelity and to internal ribosome stabilization^35^. In addition, bL31A has been reported to be important for spore germination in *Bacillus subtilis* which like *E. coli* has two bL31 paralogs, exponential phase specific bL31A and stationary phase specific bL31B^36^.

bL31B seems to play an important role in curli production needed for attachment to surfaces and biofilm formation, i.e. transition from planktonic cells to biofilm in enterohemorrhagic *E. coli*^37^. However, these studies lack systematic comparison of bL31 paralogs in the context of cell physiology and translation.

Here, we have elucidated the role of bL31A and bL31B on bacterial growth phenotype and translation reading frame maintenance. Spot-tests under various temperatures and growth competition assay demonstrated that bL31A gives growth advantage over bL31B at lower temperatures. In addition, ribosomes with bL31A maintain translation reading frame more efficiently than the bL31B containing ribosomes in vivo. Finally, we demonstrate that bL31A confers growth advantage during fast growth conditions.

## Results and discussion

### bL31A and bL31B are important but not equivalent for optimal growth at lower temperatures

Our previous study has shown that bL31 is necessary for effective cell growth under lower temperatures and for efficient translation^34^. We aim to find out whether bL31A or bL31B confers phenotypic differences during cell growth. To clarify this, the growth of *E. coli* strains expressing both bL31 paralogs (the wild type strain MG), none of the bL31 paralogs (double deletion strain ΔAB) and either bL31A or bL31B (A-strain, B-strain, respectively) was monitored at various temperatures. To ensure comparable expression of bL31A or bL31B two strains were constructed in the ΔAB strain background utilizing the conditional-replication, integration, and modular (CRIM) plasmids^38^. This approach resulted in the A-strain and the B-strain where *rpmE* (encoding bL31A) or *ykgM* (bL31B) respectively are located and expressed in the same chromosomal context as confirmed by PCR analysis and sequencing the DNA region around the insertion site.

**Figure 1:**
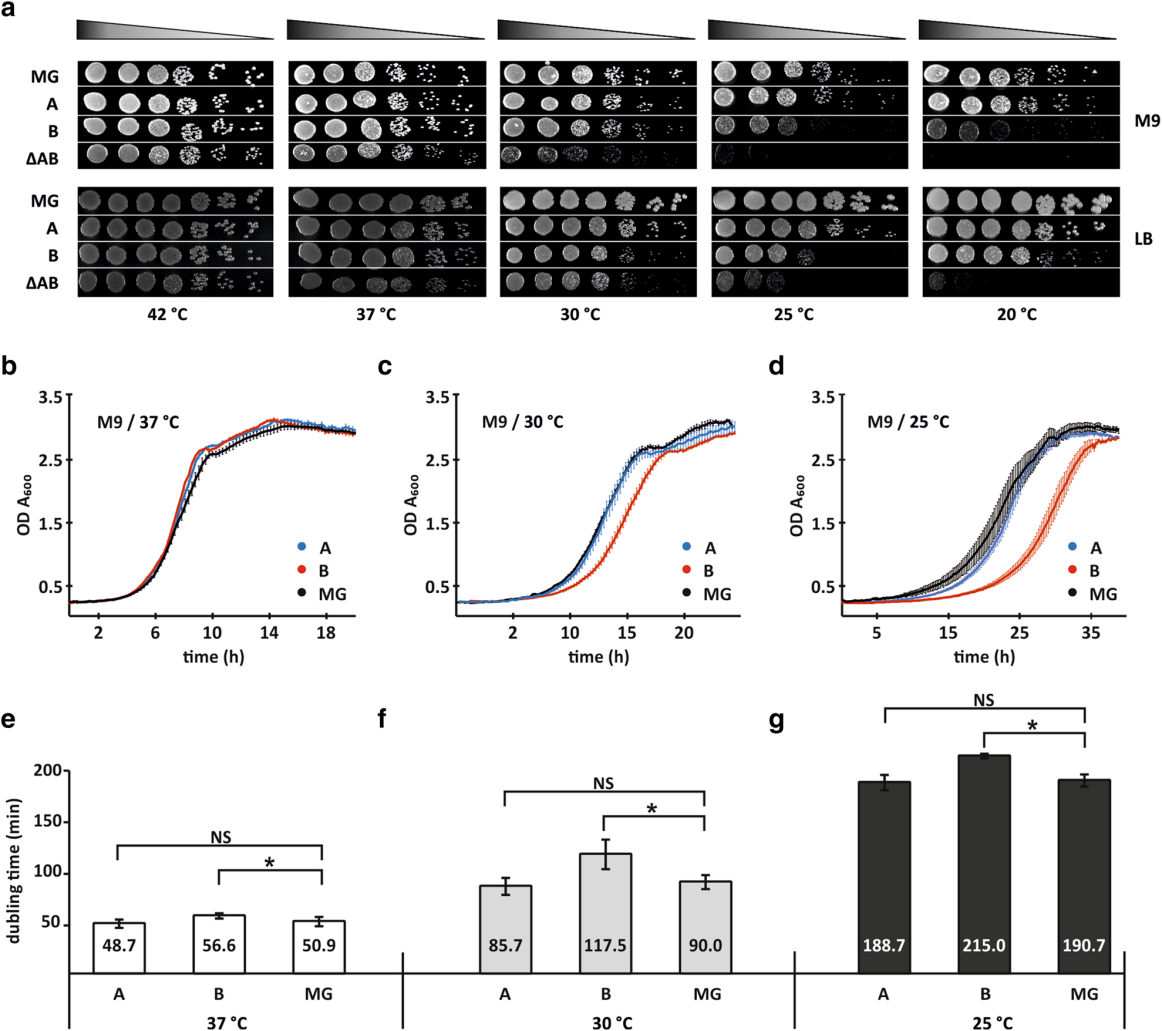
Growth phenotypes of *E. coli* strains expressing bL31 paralogs. (**a**) Serial dilutions of the wild-type (MG), bL31 deletion strain (ΔAB) and bL31 deletion strain expressing bL31A or bL31B (A, B, respectively) were spotted onto M9 medium supplemented with 0.4% glucose or LB medium and incubated at the indicated temperatures for 22–90 h (M9) or 12–60 h (LB). This experiment was done in three biological replicates and one representative biological replicate is presented. Every image was combined from rows from the single plate (Supplementary Fig. S1). (**b**–**d**) Growth curves of the A-strain, the B-strain and MG strain. Solid lines represent the average growth curve of the triplicates with ± SEM (blue the A-strain; red the B-strain and black MG strain) measured at 37 °C, 30 °C and 25 °C in M9 minimal media supplemented with 0.4% glucose. (**e**–**g**) Doubling times (min) of the A-, B- and MG strains are indicated. Statistical significance was determined by the unpaired two sample Student's *t* test (*P < 0.05; NS, not significant).

At first, we performed serial dilutions spot tests at different temperatures on rich and minimal media. At 42 °C, all strains exhibit similar growth in M9 media (Fig. 1a; Supplementary Fig. S1). At 37 °C, the A- and B-strains demonstrate growth similar to the wild type strain whereas the ΔAB strain grows slower than the wild type strain (Fig. 1a; Supplementary Fig. S1). This effect is even more pronounced at 30 °C. At 25 °C and 20 °C, the A-strain grows like the wild type strain whereas the B-strain grows weaker than the wild type strain. In the absence of bL31 cells are barely able to grow at lower temperatures. Similar growth differences appear also on LB media (Fig. 1a, Supplementary Fig. S1). It is noteworthy that bL31A or bL31B is stoichiometrically present in 50S subunits purified from the 70S ribosomes A- or B-strain (Supplementary Fig. S2). Therefore, reduced growth of the B-strain is not caused by the absence of bL31B in ribosomes. Based on spot test results we conclude that the loss of both bL31 paralogs leads to cold sensitive growth phenotype that is in agreement with earlier observations^34^. Comparison of the A- and B-strains demonstrates that the presence of bL31A in ribosomes gives growth advantage over the presence of bL31B at lower temperatures.

To further clarify the role of the bL31, the effect of paralogs on bacterial growth was monitored in M9 liquid media supplemented with glucose (0.4% f.c.) at 37 °C, 30 °C and 25 °C (Fig. 1b–d). Growth curves were recorded by monitoring the turbidity of cultures using the 96-well microtiter plate reader. Plotted growth curves measured at 37 °C showed almost equal growth characteristics for all strains analysed (Fig. 1b–g). Moderate increase in doubling time of the B-strain compared to the A-strain was observed at 30 °C (117.5 min and 85.7 min respectively) and it is most prominent at 25 °C (215 min and 188.7 min respectively; Fig. 1f–g). Overall, the shorter *lag* period and faster growth on solid and liquid media both indicate that the strain encoding only bL31A has a remarkable growth advantage over the strain encoding only bL31B during lag and exponential growth phase.

### bL31A confers higher fitness to *E. coli* than bL31B during cyclic growth but not during stationary phase

**Figure 2:**
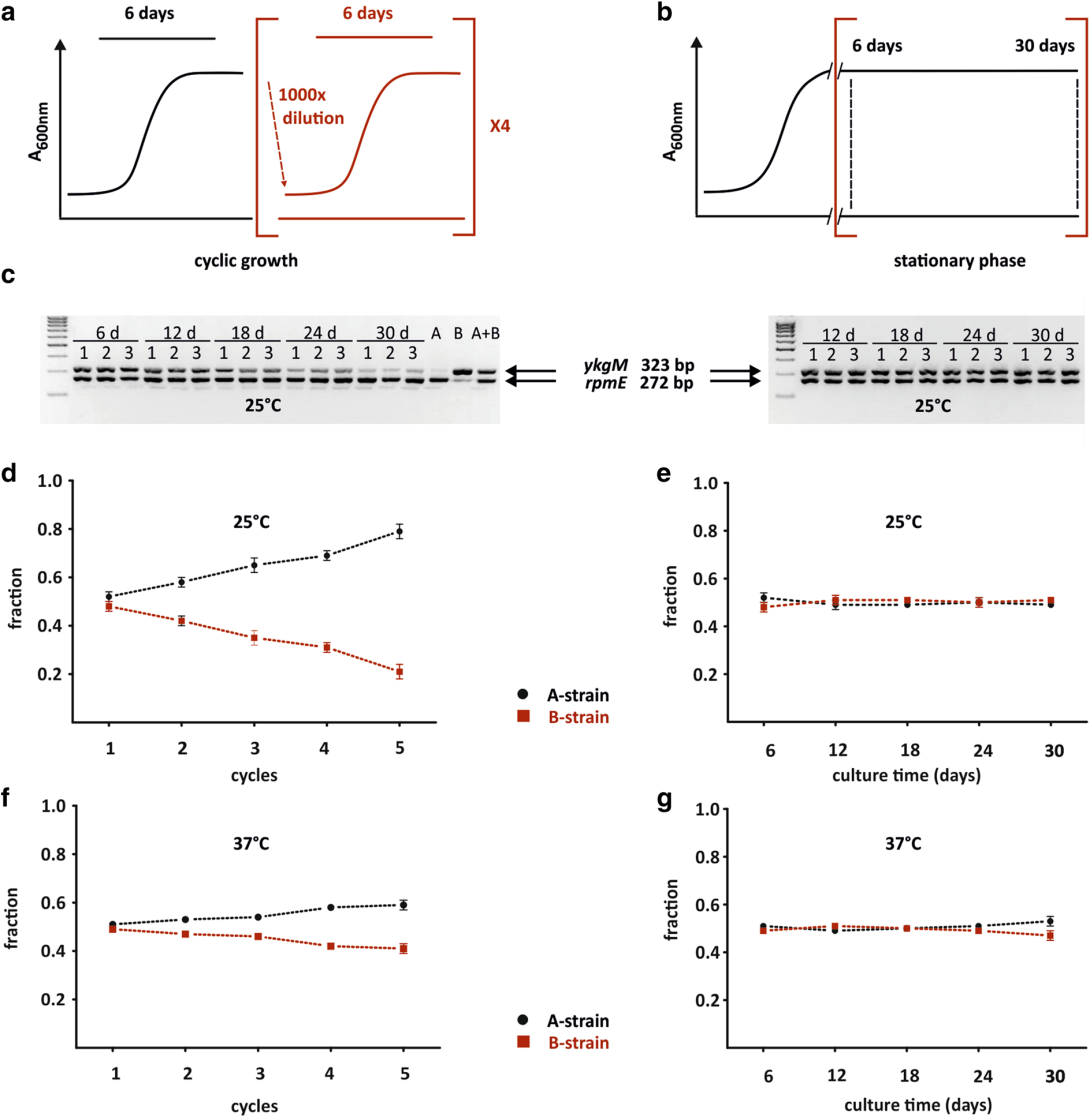
Fitness of *E. coli* expressing bL31A or bL31B is different during cyclic growth but not under stationary growth. (**a**, **b**) Experimental scheme of growth competition assay. Overnight cultures of the A and B strains were mixed at 1:1 ratio, divided into cyclic growth culture (A) and stationary phase culture (B) and cultured in M9 medium. From the stationary phase culture samples were taken after every 6 days (time points marked with dashed lines). In the case of cyclic growth samples were taken on the 6th day. Then, the same culture was diluted by 1,000 × into fresh M9 medium and grown for 6 days. Altogether this experiment lasted for 5 cycles or 30 days in case of stationary phase culture. (**c**) PCR analysis of growth competition cultures under cyclic growth (left panel) and stationary phase maintenance (right panel) at 25 °C. Purified genomic DNA from different time points (6, 12, 18, 24, 30 days) was used in the PCR reaction to detect *rpmE* and *ykgM* genes. PCR products were analyzed in 2% agarose gel and bands corresponding to *rpmE* (272 bp) and *ykgM* (323 bp) genes were quantified. Three biological replicates were analyzed. PCR reactions with genomic DNA from the A strain (lane A) or the B strain (lane B) or both strains mixed at 1:1 ratio (lane A + B) were conducted as control reactions. M-DNA size marker (**d**–**g**) Quantification of the fraction of *rpmE* and *ykgM* genes during cyclic growth (**d**, **f**) and stationary phase (**e**, **g**) at 25 °C and 37 °C. For each time point and replicate, signal intensities of bands corresponding to *rpmE* and *ykgM* genes were measured and fractions of both *rpmE* (black) and *ykgM* (red) intensities were calculated. n = 3, mean ± sd.

Fitness of the A-strain and the B-strain was compared in a growth competition assay performed at 37 °C and 25 °C. In this assay cultures of the A- and B-strains were mixed at equal ratio, separated into two batches (cyclic growth and stationary phase culture) and grown in parallel in M9 medium. After every 6 days samples were taken, and the cyclic growth culture was diluted 1,000-fold into fresh media (Fig. 2a) whereas the stationary phase culture was allowed to continue its growth in the same media (Fig. 2b). Altogether this experiment lasted for 5 cycles (30 days). In mixed cultures, the relative abundance of the A-strain and the B-strain was assessed by quantifying the fraction of the bL31A encoding gene (*rpmE*) and bL31B encoding gene (*ykgM*) from corresponding agarose gel bands (Fig. 2c; Supplementary Fig. S6). Chromosomal DNA was extracted from the samples for locus-specific PCR. Intensities of bands on agarose gel corresponding to *rpmE* and *ykgM* genes were quantified.

Under conditions of stationary phase maintenance, the fraction of the A-strain and the B-strain is approximately equal during 30 days at 25 °C and 37 °C (Fig. 2e, g). Indeed, the fraction of live cells after 16 days of incubation is approximately the same in the A- and B-strain at 37 °C or at 25 °C (Supplementary Fig. S3). By contrast, under cyclic growth conditions the fraction of the A-strain increases from 0.5 to 0.8 whereas the fraction of the B-strain decreases from 0.5 to 0.2 yielding in approximately fourfold difference after 5 cycles of growth (Fig. 2d). At 37 °C, this trend is similar but with less pronounced difference (about 1.5-fold) after 5 growth cycles (Fig. 2f). These results show that under fast growth conditions the bL31A expressing strain exhibits higher fitness than the bL31B expressing strain. These results are in agreement with growth phenotypes of the A- and B-strain on solid M9 media under 37 °C and 25 °C (Fig. 1a; Supplementary Fig. S1). We conclude that in growth competition bL31A supports bacterial cyclic growth more effectively than bL31B. Spot-tests and growth competition assay demonstrate that the growth advantage of the bL31A expressing strain over the bL31B expressing strain is manifested at lower temperatures (Figs. 1a, 2d; Supplementary Fig. S1). Notably, bL31 accelerates initiation step of translation^34^, a step that has high activation energy and is therefore temperature dependent^39^. bL31 stimulates ribosome subunit association in an initiation uncoupled reaction as well^34^.

We assumed that bL31A may facilitate 70S ribosome formation and thereby translation initiation more efficiently than bL31B. To control this possibility, the ability of bL31A or bL31B containing 50S subunits to form 70S ribosomes was tested using in vitro reassociation assay at different Mg^2+^ concentrations and sucrose density gradient analysis as described in Lilleorg et al.^34^ . Despite the fact that in the complete absence of bL31 ribosomal subunit joining is definitely influenced^34^, ribosomes containing only either bL31A or bL31B paralog demonstrated nearly identical efficiency in 70S formation (the 70S formation was unaffected when compared with control ribosomes; Supplementary Fig. S4). In addition to lower temperatures the growth advantage of the bL31A expressing strain over the bL31B expressing strain is manifested during cyclic growth. Higher fitness of the A-strain can be caused by faster adaptation to exponential growth phase and quicker growth, as suggested by shorter lag phase and doubling time of the A-strain compared to the B-strain at 30 °C and 25 °C (Fig. 1c, e and f, g). This possibility is supported by RpmE (equivalent for bL31A) being important for protein synthesis during spore germination in B. subtilis^36^. It is interesting to note that during transition from exponential to stationary phase bL31A is replaced by bL31B in wild type E. coli ribosomes^31^. bL31 may affect the rate of translation initiation. If bL31A and bL31B had a different effect it would lead to different translation initiation rate in general. bL31A is expressed during exponential growth phase^20^ when bacterial physiology is focused on rapid protein synthesis. We speculate that bL31A supports optimal translation initiation to maximize protein production during exponential phase.

### bL31 paralogs are important but not equivalent for apparent translation processivity and maintaining correct reading frame during protein synthesis

Previously, we have shown by MS that both bL31 paralogs are present in wild type ribosome population^31^. Here we focus on the role of bL31A and bL31B in reading frame maintenance by utilizing dual luciferase assay performed essentially as in^34^. For that *E. coli* strains were transformed with a plasmid encoding a fusion protein containing Renilla luciferase (Rluc) in the N-terminus and Firefly luciferase (Fluc) in the C-terminus^40^. Both activities of the fusion protein were measured from cell lysates prepared from exponentially growing cells at 30 °C. The results are expressed as the Fluc/Rluc ratio.

**Figure 3:**
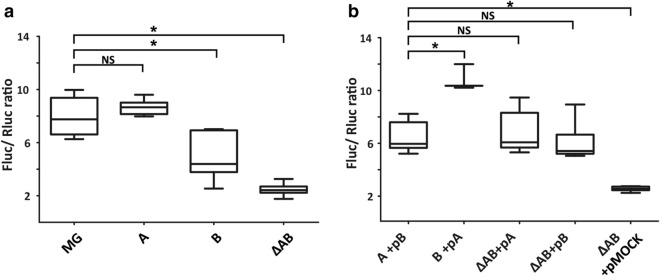
Apparent translation processivity depends on bL31 protein. Apparent translation processivity of *E. coli* strains encoding both bL31 paralogs (MG), the bL31A paralog (A), the bL31B paralog (B) or none of the bL31 paralogs (ΔAB) were assayed for luminescence with The Dual-Luciferase Reporter (DLR) Assay System (Promega)^34^. Activities of the fusion protein were measured from exponentially growing cells at 30 °C and expressed as Fluc/Rluc ratios. (**a**) Strains transformed with the dual luciferase fusion reporter, n = 9–15. (**b**) Strains containing the dual luciferase reporter complemented with a plasmid expressing bL31B (pB), bL31A (pA) or empty vector (pMOCK), n = 3–8. Statistical significance was determined by the unpaired two sample Student's *t* test (*P < 0.05; NS, not significant).

In the wild type strain, the median ratio of Fluc and Rluc activities is 7.75 (Fig. 3a). In the absence of bL31 paralogs the Fluc/Rluc ratio is 3.2 times decreased (Fig. 3a). This observation can be caused by reduced translation processivity. The overexpression of bL31A or bL31B in the ΔAB strain results in increased median Fluc/Rluc ratio (6.00, 5.35 respectively, Fig. 3b). Together, these results demonstrate that bL31 protein is important for apparent translation processivity.

Interestingly, the bL31 paralog content seems to affect the efficiency of the Rluc-Fluc translation. The A-strain displays similar results to the wild type strain (8.66 and 7.75 respectively, Fig. 3a). This can be explained by the fact that most ribosomes from the exponentially growing wild type strain contain bL31A^31^. By contrast, the B-strain exhibits about 1.8-fold decreased Fluc/Rluc ratio (4.27, Fig. 3a). This cannot be attributed to the potential lack of bL31B in ribosomes since 50S subunits purified from 70S ribosomes extracted from the exponentially growing B-strain contain bL31B protein in stoichiometric amount as validated by MS analysis (Supplementary Fig. S2). The overexpression of bL31A in the B-strain results in more than twofold increase in its Fluc/Rluc ratio leading to restauration of apparent translation processivity to the wild type level (Fig. 3b). However, when bL31B is overexpressed in the A-strain the Fluc/Rluc ratio is moderately decreased. Altogether we conclude that bL31 protein is important for determining translation processivity. Ribosomes containing bL31A appear to have slightly higher processivity in comparison with B-ribosomes.

**Figure 4:**
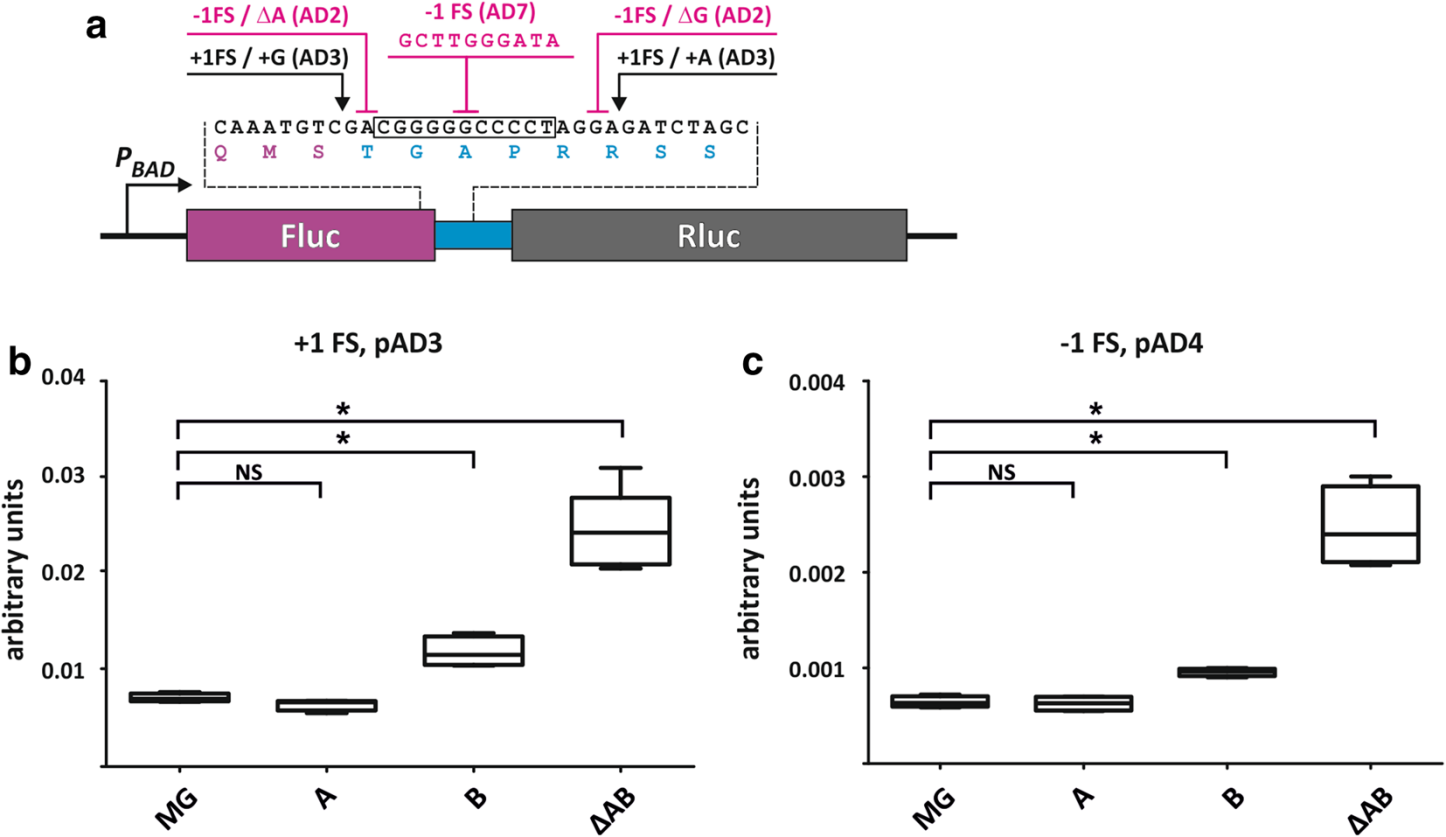
bL31A and bL31B confer different frameshifting frequency. Frameshifting levels of the same *E. coli* strains as in Fig. 3a were assayed for luminescence as in Fig. 3. (**a**) Dual luciferase reporter constructs. A gene fusion of Rluc (purple) and Fluc (grey) was used to measure FS suppression. Frameshifting sequences are indicated. The Fluc/Rluc ratios of + 1 or − 1 frameshifting reporter from every strain were divided by the average of the Fluc/Rluc ratio of the dual luciferase fusion reporter from the same strain. Results are expressed in arbitrary units. (**a**) Strains transformed with the + 1 frameshifting dual luciferase reporter (pAD3), n = 4–6. (**b**) Strains transformed with the − 1 frameshifting dual luciferase reporter (pAD4), n = 4. Statistical significance was determined by the unpaired two sample Student's *t* test (*P < 0.05; NS, not significant).

An important factor that affects translation processivity is frameshifting (FS) frequency^41^. The effect of bL31 paralogs on frameshifting frequency was analysed utilizing dual luciferase assay. Different + 1 and − 1 frameshift sites were introduced between the Rluc and Fluc gene^40^ (Fig. 4a).

First, in the wild type strain median + 1 frameshifting level is about tenfold higher than median − 1 frameshifting level (0.007 and 0.0006 Fig. 4b, c) indicating that + 1 frameshift events happen approximately 10 times more frequently than − 1 frameshift events. The A-strain displays practically the same median + 1 and − 1 frameshifting levels as the wild type strain (Fig. 4, Supplementary Fig. S5). This is in accordance with previous results^34^ and can be rationalized bearing in mind that the majority of the wild type ribosomes contains bL31A during exponential growth phase^31^. The B-strain exhibits higher median frameshifting levels in 4 out of 5 frameshift sites tested as compared to the wild type strain (Fig. 4, Supplementary Fig. S5). Comparison with the A-strain demonstrates that the B-strain exhibits 1.7 times higher median + 1 FS (Fig. 4b, Supplementary Fig. S5) and 1.6 times higher − 1 FS (Fig. 4c, Supplementary Fig. S5). In the absence of bL31 protein both + 1 and − 1 frameshifting are about 3 times increased as compared to the wild type strain (Fig. 4, Supplementary Fig. S5). This result is in line with previous research^34^.

Dual luciferase assay demonstrates that bL31 protein contributes to apparent translation processivity and maintaining translation reading frame in vivo. Ribosomes containing bL31B have reduced apparent processivity (Fig. 3a) and they are more prone to shift reading frame than bL31A ribosomes (Fig. 4). Therefore, ribosomes in the B-strain have a higher probability to encounter out-of-frame stop codon leading to premature translation termination. This was demonstrated by decreased Fluc/Rluc ratio. Reduced translation processivity correlates with increased frameshifting frequency^41^.

The aim of this study was to find out whether ribosome heterogeneity with respect to bL31A and bL31B is functional. First, wild type ribosomes are heterogeneous in vivo^31^. Second, this study provides experimental support that r-protein paralog composition (bL31A *versus* bL31B) can affect cell growth and translation outcome. bL31A containing ribosomes have higher apparent processivity and lower frameshifting level as compared to ribosomes with bL31B. The effect of various stress conditions on ribosome r-paralog composition and its possible association with translation outcome and cell physiology remains an intriguing subject for future studies.

## Methods

### Bacterial strains and plasmids

*E. coli* strains and plasmids are listed in Table 1. Bacteria were cultivated in LB medium (1% Tryptone, 0.5% yeast extract, 0.5% NaCl, and 1.5% agar), in rich 2xYT medium (10 g/l yeast extract, 16 g/l Tryptone, and 5 g/l NaCl), or in M9 minimal medium (M9 salts (22 mM KH_2_PO_4_, 47.8 mM Na_2_HPO_4_, 18.7 mM NH_4_Cl, 8.7 mM NaCl), 0.4% glucose, 2 mM MgSO_4_, 0.1 mM CaCl_2_, 0.5 μg/ml thiamine^42^ supplemented with 1.5% agarose for solid medium. For measuring frameshifting using dual luciferase assay M9 media was supplemented with 0.4% sorbitol instead of glucose and with antibiotics as indicated.

**Table 1:** Bacterial strains and plasmids.

Strain or plasmid	Genotype or characteristics	Source or reference
*Strains*		
MG1655	F-, l-, rph-1	Laboratory stock
A-strain	MG1655 *ΔykgMΔykgOΔrpmE::kan* in ΔlacIΔlacZ background; chromosomal expression of *rpmE* under tac promoter	This study
B-strain	MG1655 *ΔykgMΔykgOΔrpmE::kan* in ΔlacIΔlacZ background; chromosomal expression of *ykgM* under tac promoter	This study
ΔAB strain	MG1655*ΔrpmEΔykgM::kan*	^34^
MGZI	MG1655*ΔlacIΔlacZ::kan*	^34^
MGZI-ΔAB	MG1655*ΔlacIΔlacZΔrpmEΔykgM::kan*	^34^
*Plasmids*		
pHB	A derivate of pHSG576; araC promoter region from pBADMycHisC vector	This study
pHB-Rluc-Fluc	Low copy expression of the Rluc-Fluc gene; Cm^R^	This study
pHB-AD5	Reporter plasmid with a + 1 frameshift signal between the Rluc and Fluc gene, Cm^R^	This study
pHB-AD3	Reporter plasmid with a + 1 frameshift signal between the Rluc and Fluc gene, Cm^R^	This study
pHB-AD2	Reporter plasmid with a − 1 frameshift signal between the Rluc and Fluc gene, Cm^R^	This study
pHB-AD4	Reporter plasmid with a − 1 frameshift signal between the Rluc and Fluc gene, Cm^R^	This study
pHB-AD7	Reporter plasmid with a − 1 frameshift signal between the Rluc and Fluc gene, Cm^R^	This study
pBT (pMOCK)	tac promoter; derivate of pBADMycHisC; Amp^R^, vector for experimental control	^34^
pBT-bL31A	pBT-*rpmE*, Amp^R^, high copy expression of bL31A	^34^
pBT-bL31BbL36B	pBT-*ykgMykgO*, Amp^R^, high copy expression of bL31B and bL36B	This study

### Construction of strains and plasmids

For comparable expression of *rpmE* (encoding bL31A) or *ykgM* (encoding bL31B) the A-strain and the B-strain were constructed. Briefly, *rpmE* or *ykgM* genes were first cloned into the pAH55 plasmid (a conditional-replication, integration, and modular CRIM plasmid, gene expression is under the control of tac promoter^38^). Then, plasmids were integrated into the genome of the ΔAB strain using conditional-replication and integration approach^38^.

For the low copy expression of Rluc-Fluc fusion protein in dual luciferase assay a derivate of pHSG576^34^ referred to as pHB was used. The pHB vector was constructed by replacing the tac promoter region from pDR540 (Pharmacia) with arabinose-inducible araBAD promoter and araC gene region of pBADMycHisC vector using SphI-BamHI restriction enzymes.

Genes encoding Renilla luciferase and Firefly luciferase in the same open reading frame (Rluc-Fluc)^43^ were cloned into the pHB low-copy expression vector. In addition, Rluc-Fluc constructs containing different frameshift signals in the linker region between the Rluc and Fluc genes^40^ were cloned into the pHB vector. The nomenclature of the generated plasmids corresponds to that of Devaraj and colleges’^40^. All constructed plasmids were verified by DNA sequencing. For DNA manipulations, plasmid DNA isolation and transformation standard techniques were used^44^.

For the expression of bL31B *ykgMykgO* fragment was amplified and cloned into the high copy plasmid pBT as specified in^34^.

### Temperature sensitivity assay

Bacteria from MG1655, A-strain, B-strain and ΔAB strain were grown in LB or M9 medium supplemented with 0.4% glucose at 37 °C overnight. Cultures were serially diluted (10^6^–10^3^ cells/ml), spotted on LB or M9 medium plates, and incubated at 42 °C, 37 °C, 30 °C, 25 °C, 20 °C.

### Growth curve measurements

Cultures were grown overnight and diluted into fresh M9 medium supplemented with 0.4% glucose (starting OD A_600_ 0,004). The aliquots of 125 μl cultures were incubated in a Polarstar Omega 96-well plate reader at 37 °C, 30 °C and 25 °C and the turbidity of bacterial culture (A_600_) was monitored at 7 min intervals. For each strain, generation times for at least three biological replicas with two technical replicas were determined and presented as mean values with SEM. Statistical significance was determined by the unpaired two sample Student's t test at a significance level of 0.05.

### Dual luciferase assay

Frameshifting was assayed by dual luciferase assay using MG, the A, the B and ΔAB strains in *ΔlacIΔlacZ* background transformed with pHB plasmids expressing Rluc-Fluc construct variants (Table 1). Bacterial cells were grown in M9 medium supplemented with 0.4% sorbitol (weight/ volume) and chloramphenicol (final concentration 10 μg/ml) at 30 °C overnight. Overnight cultures were diluted to A_600_ 0.1 in the fresh M9 medium without chloramphenicol (final volume 3 ml) and grown at 30 °C to mid-logarithmic phase (A_600_ 0.4). Samples (1 ml) were taken, cells were collected by centrifugation (4,000 rpm, 4 min, 4 °C), flash-frozen in liquid nitrogen and kept under − 80 °C until bioluminescent measurements with Dual-Luciferase Reporter (DLR) Assay System. The expression of Rluc–Fluc fusion protein was induced by adding 10 μl arabinose (final concentration 0.2%) to 1 ml of the remaining culture. After 1 h of induction at 30 °C cells were processed as described above for pre-induction samples. Frozen cells were resuspended in 400 μl Passive Lysis Buffer of Dual-Luciferase Reporter Assay System and incubated on ice for 10 min. Next, 40 μl of the extract was assayed for Firefly and Renilla luciferase as described in Lilleorg et al. 2017. Fluc activity (relative units) was calculated as the ratio of the Fluc to Rluc activity. Statistical significance was determined by the unpaired two sample Student's t test at a significance level of 0.05.

### Growth competition assay

The A strain and the B-strain were cultured in M9 medium^42^ supplemented with 0.4% glycose at 37 °C or 25 °C overnight. Overnight cultures of both strains were mixed together at 1:1 ratio (A_260_) into 40 ml fresh M9 medium (starting A_260_ 0.1). Starting culture was divided into two batches (both 20 ml) and grown at specified temperature in a shaker. From one batch (stationary growing culture) samples (1 ml) were taken after every 6 days, flash frozen in liquid nitrogen and kept at − 80 °C until further analysis. From the second batch (cyclic growing culture) a sample was taken on the 6th day as specified above. Then, the same culture was diluted by 1,000 × into the fresh M9 medium and grown at specified temperature for 6 days. Altogether samples from 5 consecutive 6-day cycles were obtained.

From all samples genomic DNA was purified by GeneJET Genomic DNA Purification Kit (Thermo Scientific) according to manufacturer’s protocol. Genomic DNA (20 ng) was subjected to PCR to detect the proportion of genes encoding bL31A or bL31B (*rpmE* and *ykgM*, respectively) in mixed *E. coli* populations. For that, primers specific for regions flanking λ att sites were used: DIR 5′-GCGGATAACAATTTCACACAGGAAACAG-3′ and REV 5′-TTCCCATATGGTACCAGCTGCAG-3′. PCR reaction products were analyzed in 2% agarose gel and quantified with UVITec1D software. Signal intensities of PCR products corresponding to *rpmE* (272 bp encoding bL31A protein) or *ykgM* (323 bp encoding bL31B protein) were measured and quantified with FireReader V10 (Uvitec). Fractions of corresponding to both *rpmE* and *ykgM* were calculated: *rpmE*/ *rpmE* + *ykgM* and *ykgM/ykgM* + *rpmE*. Altogether three biological replicates were analyzed.

## Supplementary information

Supplementary information.

## Data Availability

The mass spectrometry proteomics data have been deposited to the ProteomeXchange Consortium via the PRIDE partner repository with the dataset identifier PXD018123 and 10.6019/PXD018123.

## References

[1] Duval M, Simonetti A, Caldelari I, Marzi S (2015). Multiple ways to regulate translation initiation in bacteria: mechanisms, regulatory circuits, dynamics. Biochimie.

[2] Sonenberg N, Hinnebusch AG (2009). Regulation of translation initiation in eukaryotes: mechanisms and biological targets. Cell.

[3] Ferretti MB, Barre JL, Karbstein K (2018). Translational reprogramming provides a blueprint for cellular adaptation. Cell Chem. Biol..

[4] Genuth NR, Barna M (2018). The discovery of ribosome heterogeneity and its implications for gene regulation and organismal life. Mol. Cell.

[5] Byrgazov K, Vesper O, Moll I (2013). Ribosome heterogeneity: another level of complexity in bacterial translation regulation. Curr. Opin. Microbiol..

[6] Xue S, Barna M (2012). Specialized ribosomes: a new frontier in gene regulation and organismal biology. Nat. Rev. Mol. Cell Biol..

[7] Dinman JD (2016). Pathways to specialized ribosomes: the brussels lecture. J. Mol. Biol..

[8] Gilbert WV (2011). Functional specialization of ribosomes?. Trends Biochem. Sci..

[9] Ferretti MB, Karbstein K (2019). Does functional specialization of ribosomes really exist?. RNA.

[10] Mauro VP, Edelman GM (2002). The ribosome filter hypothesis. PNAS.

[11] Emmott E, Jovanovic M, Slavov N (2019). Ribosome stoichiometry: from form to function. Trends Biochem. Sci..

[12] Kurylo CM (2018). Endogenous rRNA sequence variation can regulate stress response gene expression and phenotype. Cell Rep..

[13] Song W (2019). Divergent rRNAs as regulators of gene expression at the ribosome level. Nat. Microbiol..

[14] Baldridge KC, Contreras LM (2014). Functional implications of ribosomal RNA methylation in response to environmental stress. Crit. Rev. Biochem. Mol. Biol..

[15] Nesterchuk MV, Sergiev PV, Dontsova OA (2011). Posttranslational modifications of ribosomal proteins in *Escherichia coli*. Acta Naturae.

[16] Shi Z, Barna M (2015). Translating the genome in time and space: specialized ribosomes, RNA regulons, and RNA-binding proteins. Annu. Rev. Cell Dev. Biol..

[17] Sauert M, Temmel H, Moll I (2015). Heterogeneity of the translational machinery: variations on a common theme. Biochimie.

[18] Yutin N, Puigbò P, Koonin EV, Wolf YI (2012). Phylogenomics of prokaryotic ribosomal proteins. PLoS ONE.

[19] Makarova KS, Ponomarev VA, Koonin EV (2001). Two C or not two C: recurrent disruption of Zn-ribbons, gene duplication, lineage-specific gene loss, and horizontal gene transfer in evolution of bacterial ribosomal proteins. Genome Biol.

[20] Nanamiya H (2004). Zinc is a key factor in controlling alternation of two types of L31 protein in the *Bacillus subtilis *ribosome. Mol. Microbiol..

[21] Natori Y (2007). A fail-safe system for the ribosome under zinc-limiting conditions in *Bacillus subtilis*. Mol. Microbiol..

[22] Dow A, Prisic S (2018). Alternative ribosomal proteins are required for growth and morphogenesis of Mycobacterium smegmatis under zinc limiting conditions. PLoS ONE.

[23] Gabriel SE, Helmann JD (2009). Contributions of Zur-controlled ribosomal proteins to growth under zinc starvation conditions. J. Bacteriol..

[24] Akanuma G, Nanamiya H, Natori Y, Nomura N, Kawamura F (2006). Liberation of zinc-containing L31 (RpmE) from ribosomes by Its paralogous gene product, YtiA in *Bacillus subtilis*. J. Bacteriol..

[25] Nanamiya H, Kawamura F (2010). Towards an elucidation of the roles of the ribosome during different growth phases in *Bacillus subtilis*. Biosci. Biotechnol. Biochem..

[26] Li Y (2018). Zinc depletion induces ribosome hibernation in mycobacteria. PNAS.

[27] Chen, Y.-X. *et al.* Selective translation by alternative bacterial ribosomes. https://www.biorxiv.org/content/10.1101/605931v2 (2019).

[28] Hensley MP (2012). Characterization of Zn(II)-responsive ribosomal proteins YkgM and L31 in *E. coli*. J. Inorganic Biochem..

[29] Graham AI (2009). Severe zinc depletion of escherichia coli: roles for high affinity zinc binding by ZinT, Zinc transport and zinc-independent proteins. J. Biol. Chem..

[30] Aseev LV, Boni IV (2011). Extraribosomal functions of bacterial ribosomal proteins. Mol. Biol..

[31] Lilleorg S (2019). Bacterial ribosome heterogeneity: changes in ribosomal protein composition during transition into stationary growth phase. Biochimie.

[32] Fischer N (2015). Structure of the *E. coli* ribosome-EF-Tu complex at <3 A resolution by Cs-corrected cryo-EM. Nature.

[33] Jenner L, Demeshkina N, Yusupova G, Yusupov M (2010). Structural rearrangements of the ribosome at the tRNA proofreading step. Nat. Struct. Mol. Biol..

[34] Lilleorg S, Reier K, Remme J, Liiv A (2017). The intersubunit bridge B1b of the bacterial ribosome facilitates initiation of protein synthesis and maintenance of translational fidelity. J. Mol. Biol..

[35] Chadani Y (2017). Intrinsic ribosome destabilization underlies translation and provides an organism with a strategy of environmental sensing. Mol. Cell.

[36] Sinai L, Rosenberg A, Smith Y, Segev E, Ben-Yehuda S (2015). The Molecular timeline of a reviving bacterial spore. Mol. Cell.

[37] Lim J (2011). YkgM and ZinT proteins are required for maintaining intracellular zinc concentration and producing curli in enterohemorrhagic *Escherichia coli* (EHEC) O157:H7 under zinc deficient conditions. Int. J. Food Microbiol..

[38] Haldimann A, Wanner BL (2001). Conditional-replication, integration, excision, and retrieval plasmid-host systems for gene structure-function studies of bacteria. J. Bacteriol..

[39] McCarthy JEG, Gualerzi C (1990). Translational control of prokaryotic gene expression. Trends Genet..

[40] Devaraj A, Shoji S, Holbrook ED, Fredrick K (2009). A role for the 30S subunit E site in maintenance of the translational reading frame. RNA.

[41] Dong H, Kurland CG (1995). Ribosome mutants with altered accuracy translate with reduced processivity. J. Mol. Biol..

[42] Sambrook J, Russell D (2001). Molecular Cloning: A Laboratory Manual.

[43] Harger JW, Dinman JD (2003). An in vivo dual-luciferase assay system for studying translational recoding in the yeast *Saccharomyces cerevisiae*. RNA.

[44] Green MR, Sambrook J (2012). Molecular cloning: a laboratory manual.

