# Supplementary material for: Phenotypic effects of paralogous ribosomal proteins bL31A and bL31B in E. coli
Source: Sci Rep. 2020 Jul 15;10:11682. doi: 10.1038/s41598-020-68582-2 (PMC7363858; doi:10.1038/s41598-020-68582-2)
Supplement: Supplementary file 1 — Supplementary information. [file 41598_2020_68582_MOESM1_ESM.pdf]

Phenotypic effects of paralogous ribosomal proteins bL31A and bL31B in *E. coli*

Silva Lilleorg, Kaspar Reier, Pavel Volõnkin, Jaanus Remme, Aivar Liiv*

Institute of Molecular and Cell Biology, University of Tartu, Riia 23B, Tartu 51010, Estonia

*Corresponding author:

Aivar Liiv

Email: aliiv@ut.ee

Riia street 23B, Tartu, 51010, Estonia

SUPPLEMENTARY INFORMATION

List of Supplementary Materials:

Supplementary Figure 1.

Growth phenotypes of *E. coli* strains expressing bL31 paralogs.

Supplementary Figure 2.

Quantification of ribosomal large subunit proteins in the 70S ribosomes of the A-strain and B-strain.

Supplementary Figure 3.

Fraction of live bacterial cells is stable during 16 days.

Supplementary Figure 4.

Analysis of 70 S formation *in vitro*.

Supplementary Figure 5.

bL31A and bL31B confer different frameshifting frequency.

Supplementary Figure 6.

PCR analysis of growth competition cultures (the original unprocessed image).

Supplementary Methods

Supplementary Figure S1.

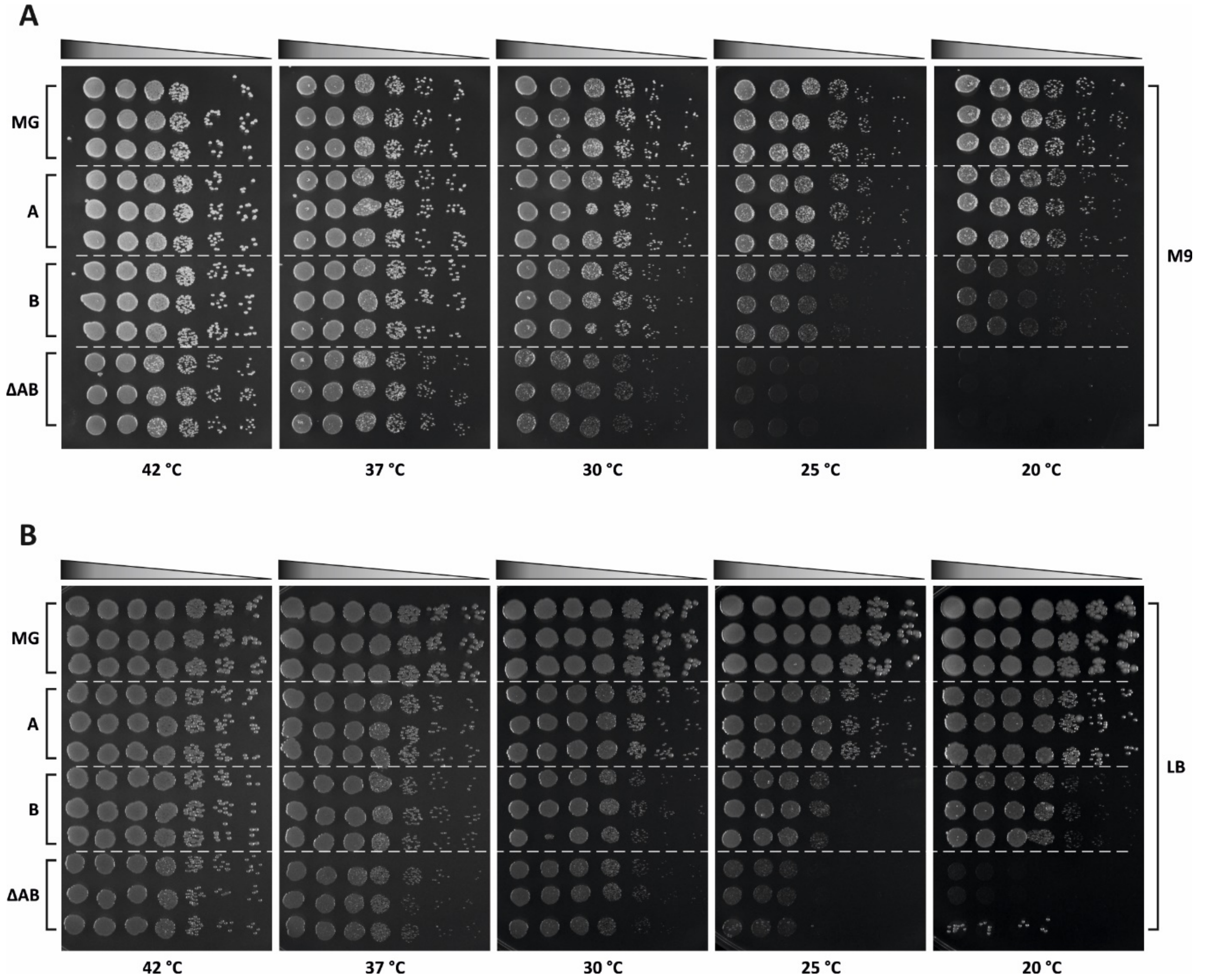

Growth phenotypes of *E. coli* strains expressing bL31 paralogs. Original images of the plates. Sections of the each of the plates were used in main Figure.1a. MG1655, A-strain, B-strain and ΔAB strain grown in LB or M9 solid growth medium supplemented with 0.4% glucose. Overnight cultures were grown in M9 (panel A) or LB medium (panel B), serially diluted (10^6 – 10^3 cells/ml), spotted on LB or M9 medium plates, and incubated at 42°C, 37°C, 30°C, 25°C, 20°C. Three biological replicates of each strain were analyzed on the same plate.

Supplementary Figure S2.

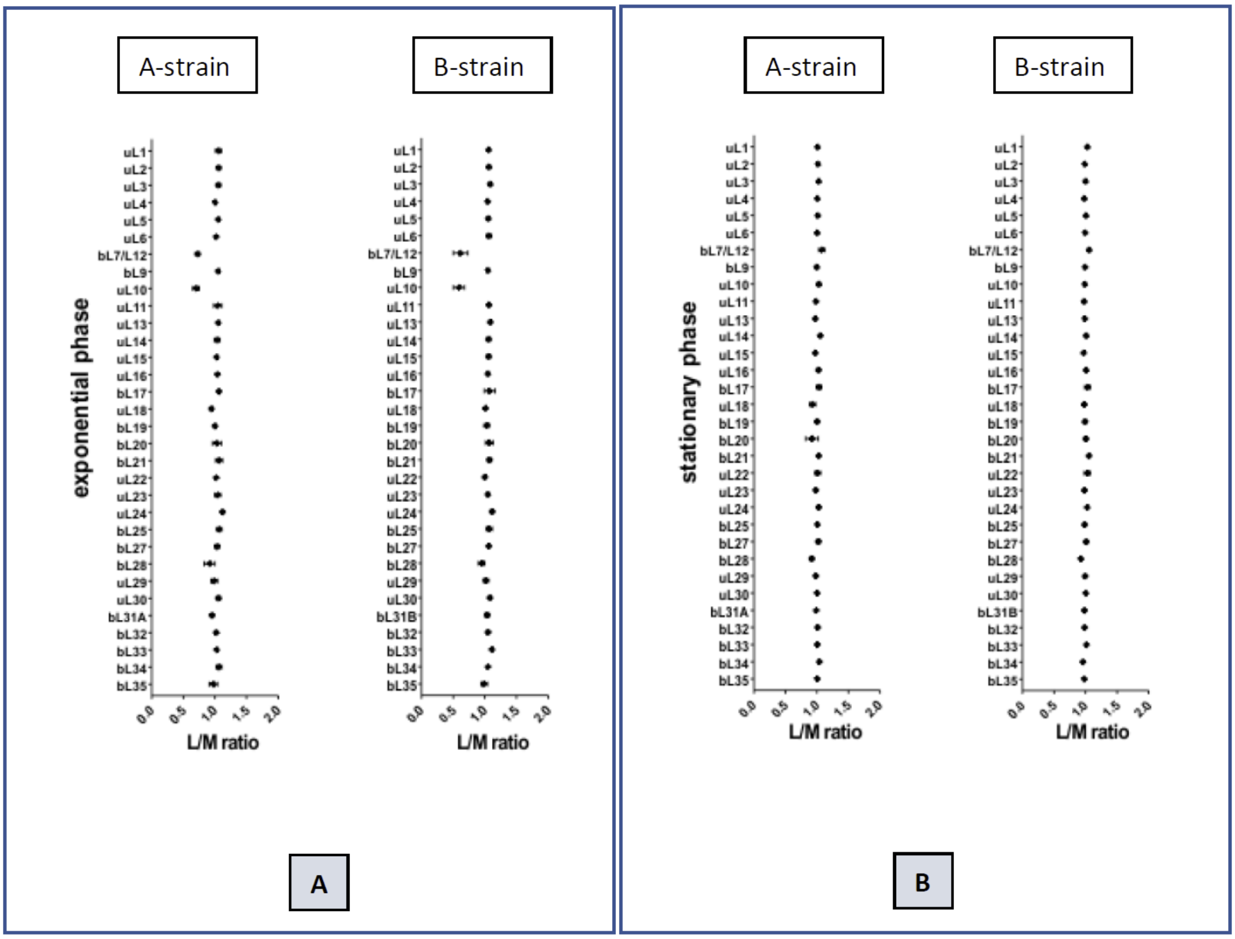

Quantification of ribosomal large subunit proteins in the 70S ribosomes of the A-strain and B-strain. Ribosomes were purified from cells collected at (A) exponential (A600 1) and (B) stationary growth phase (24h post inoculation) and mixed in 1:1 molar ratio with standard 70S ribosomes containing medium-heavy labeled arginine ($[^{13}C]_6H_{14}N_4O_2$) and lysine ($C_6[^2H]_4H_{10}N_2O_2$). Protein quantities except for bL36 were determined by quantitative mass-spectrometry. Light to medium-heavy (L/M) ratios were calculated from MS data for each protein. Data from two biological replicates is presented as mean with standard error.

Supplementary Figure S3.

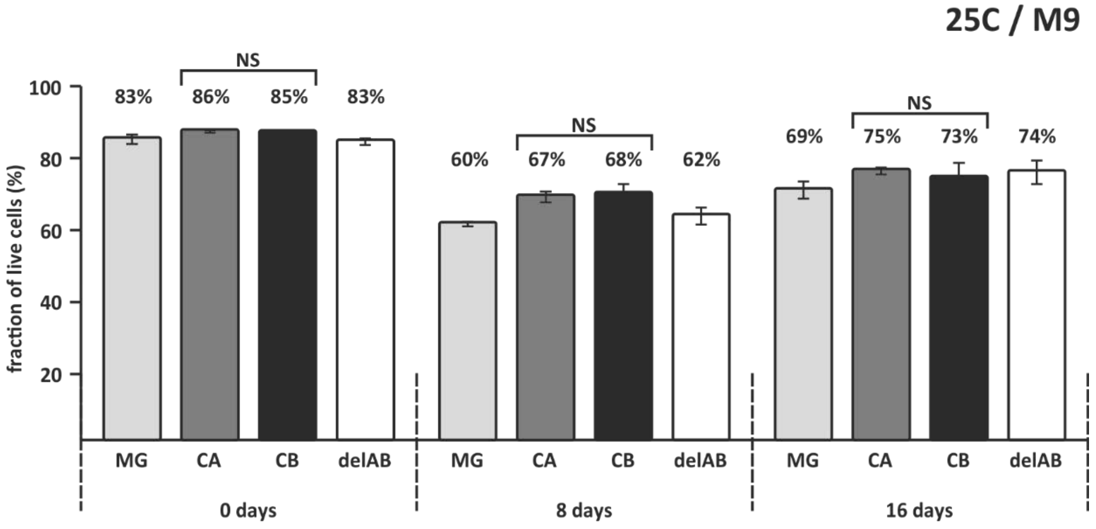

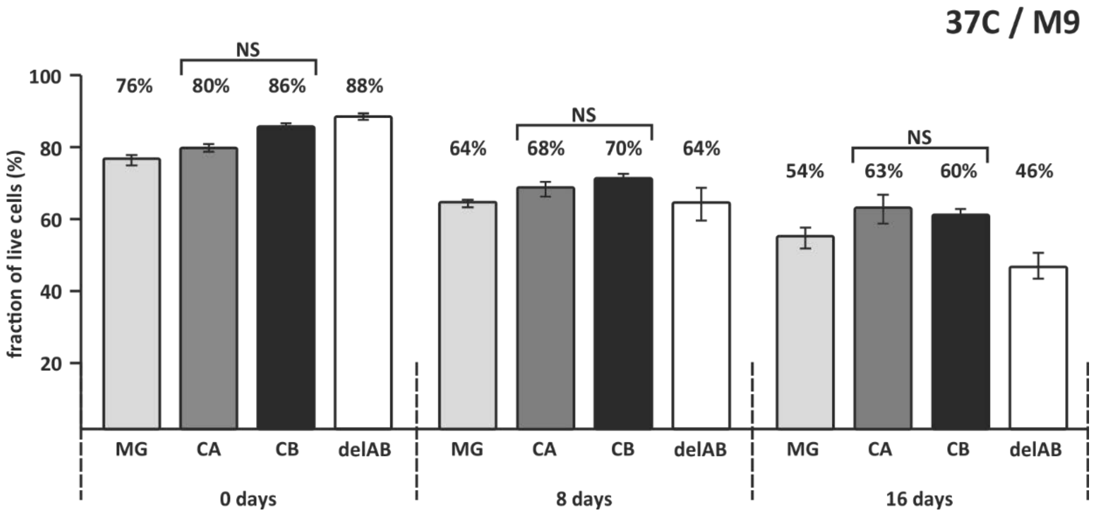

Fraction of live bacterial cells is stable during 16 days. Wild-type (MG), bL31 deletion strain (ΔAB) and bL31 deletion strain expressing bL31A or bL31B (A, B, respectively) were grown separately in M9 medium supplemented with 0.4% glucose at 37°C (upper panel) or 25°C (lower panel). On the 0th, 8th and 16th day samples were taken, stained with SYTO 9 and propidium iodide (LIVE/DEAD BacLight Bacterial Viability Kit) and analyzed with fluorescence-activated cell sorter. Fraction of live cells was calculated by dividing the number of live cells by the number of dead and live cells. Means ± SEM of at least four biological replicates are presented. Statistical significance was determined by the unpaired two sample Student's *t* test (**P* < 0.05; NS, not significant).

Supplementary Figure S4 (2/1)

A

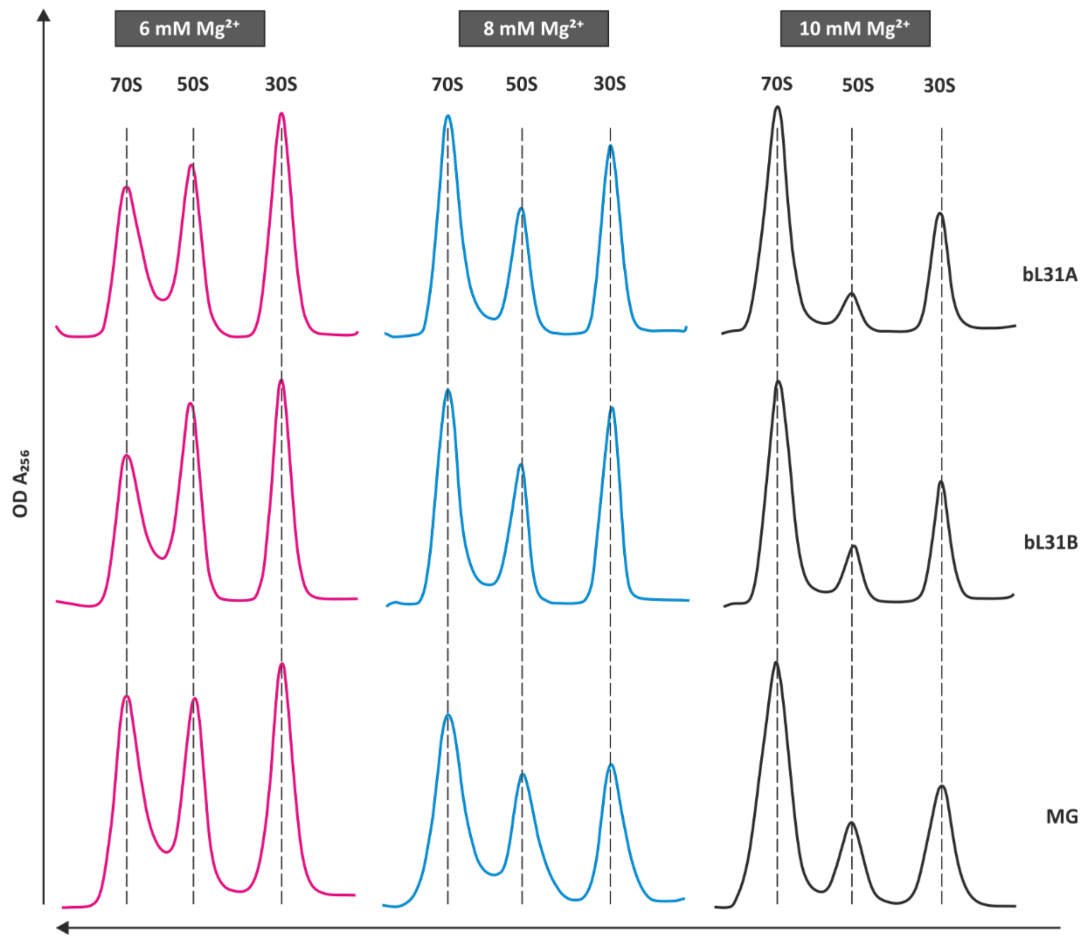

B

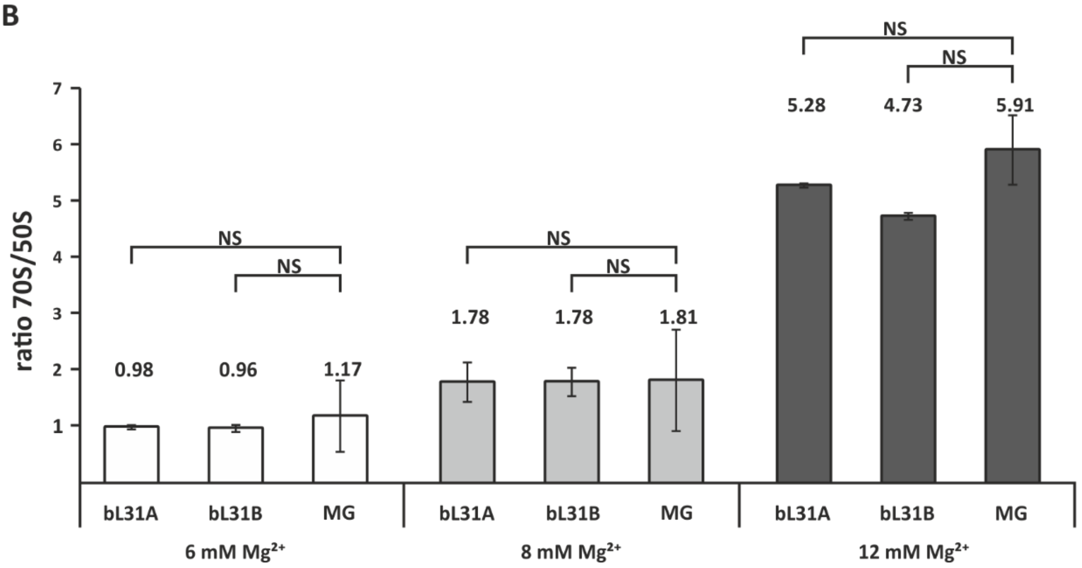

Supplementary Figure S4 (2/2)

Analysis of 70 S formation in vitro. 5 A260 units of 50S subunits were mixed with 5 A260 units of wild-type 30 S subunits and incubated at 37 °C for 30 min before being loaded onto 10–25% sucrose gradients with appropriate $MgSO_4$ concentrations, followed by ultracentrifugation. Three different $MgSO_4$ concentrations: 6mM (red), 8mM (blue) and 10mM (black) were used for association analysis. The association of wild type exponential phase specific 50S and 30S subunits under these assay conditions was used as a control. The direction of sedimentation is from right to left. The peaks of 70S and free 50S and 30S are marked by dotted lines. (A) Representative association profiles in one of three independent experiments are shown for bL31A (upper panel), bL31B (middle panel) and MG ribosomes (MG=wild type; lower panel). (B) 70S/50S ratios of association reactions. Areas under 70S and 50S were quantified using ImageJ image processing software and 70S/50S ratios were calculated. The average (mean ± SEM) ratios of at least three biological replicates are plotted. Statistical significance was determined by the unpaired two sample Student's *t* test (*$P < 0.05$; NS, not significant).

Supplementary Figure S5.

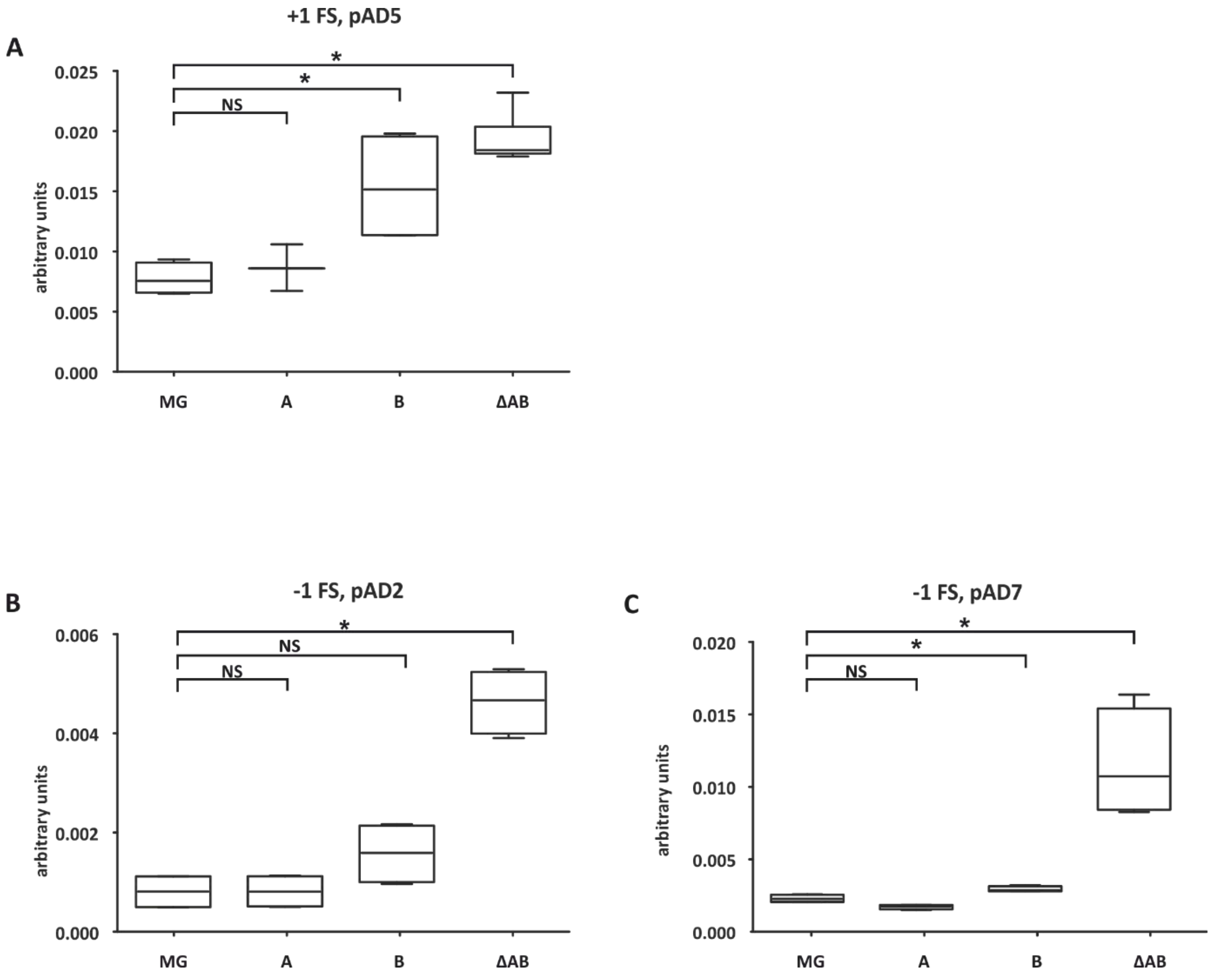

bL31A and bL31B confer different frameshifting frequency. Frameshifting levels of the same *E. coli* strains as in Fig 3A were assayed for luminescence as in Fig 3. The Fluc/Rluc ratios of +1 or -1 frameshifting reporter from every strain were divided by the average of the Fluc/Rluc ratio of the 0 frame reporter from the same strain. Results are expressed in arbitrary units. This figure belongs to Fig 4. (a) Strains transformed with the +1 frameshifting dual luciferase reporter (pAD5), n = 3-6. (b and c) Strains transformed with the -1 frameshifting dual luciferase reporter (pAD2, pAD7), n = 4.

Supplementary Figure S6.

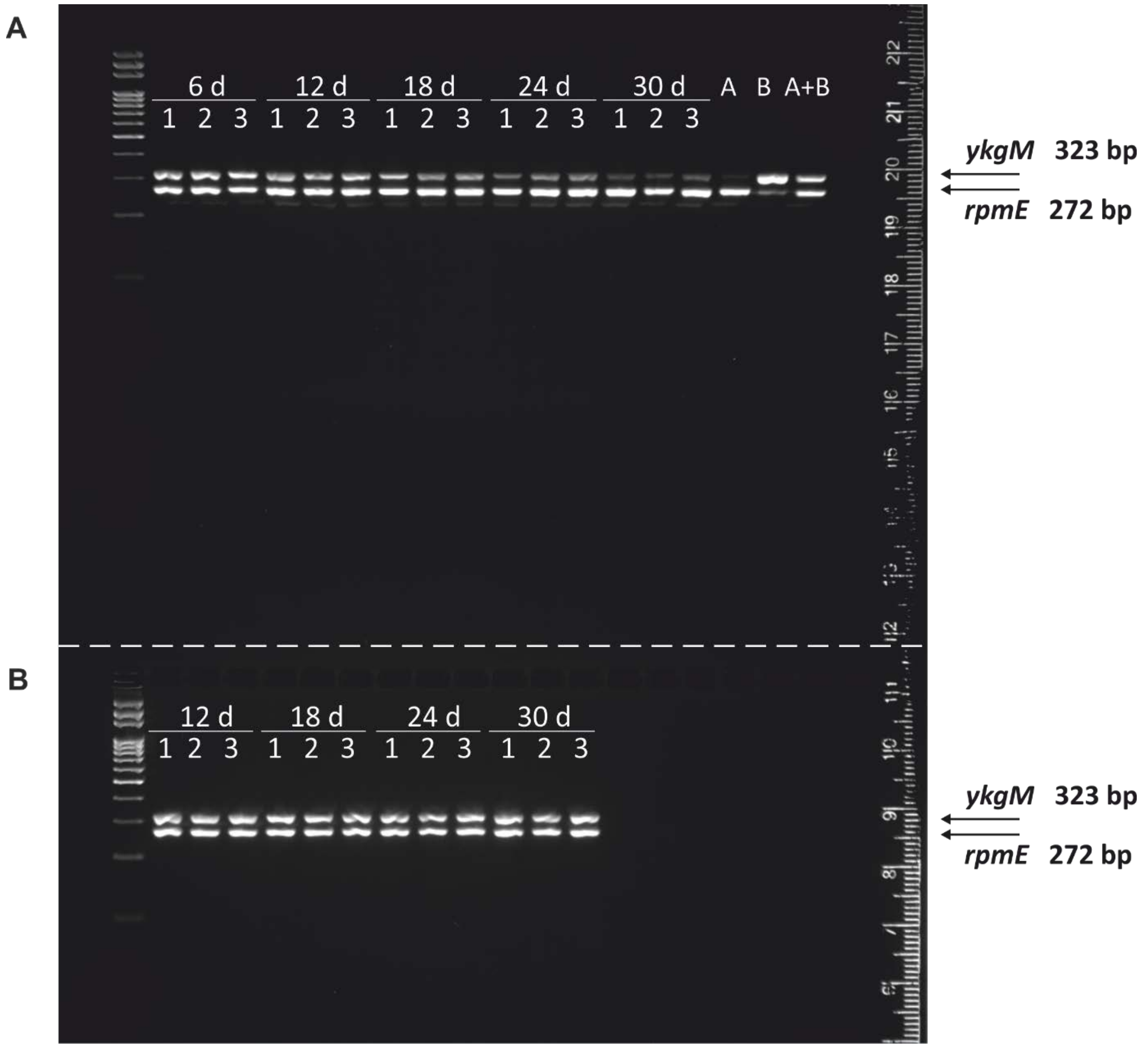

PCR analysis of growth competition cultures. Original unprocessed image of the gel (main figure 2). PCR analysis under cyclic growth (A) and stationary phase maintenance (B) at 25°C. Purified genomic DNA from different time points (6, 12, 18, 24, 30 days) was used in the PCR reaction to detect *rpmE* and *ykgM* genes. PCR products were analyzed in 2% agarose gel and bands corresponding to *rpmE* (272 bp) and *ykgM* (323 bp) genes were quantified. PCR reactions with genomic DNA from the A strain (lane A) or the B strain (lane B) or both strains mixed at 1:1 ratio (lane A+B) were conducted as control reactions.

Supplementary methods

Growth analysis on LB or M9 solid growth medium

Overnight cultures were grown in LB or M9 medium supplemented with 0.4% glucose, serially diluted (10^6 – 10^3 cells/ml), spotted on LB or M9 medium plates, and incubated at 42°C, 37°C, 30°C, 25°C, 20°C. Three biological replicates of each strain were analyzed.

Mass-spectrometry analysis: cell growth, purification of ribosomes, and sample preparation

The A-strain and B-strain were grown at 37°C in MOPS medium [1] supplemented with 0.2% glycose and containing unlabeled arginine and lysine until exponential (3 hours, A600 1) or stationary (24 hours) growth phase. 70S ribosomes were purified from cells essentially as in [2]. However, after separation by sucrose gradient centrifugation 70S ribosomes were not pelleted. They were mixed in equimolar ratio with standard 70S ribosomes that were uniformly labelled with medium-heavy arginine ($[_{13}C]_6H_{14}N_4O_2$) and lysine ($C_6[_2H]_4H_{10}N_2O_2$) and prepared as described [3]. For quantification of r-proteins the same standards were used as in [3] and samples were prepared for mass spectrometry analysis following [3].

MS data analysis

Ribosomal proteins were quantified with MaxQuant (version 1.5.6.0) and Perseus software (v.1.6.2.3. In case of bL31A and bL31B quantification was done using Skyline v.3.6 software. Search parameters were the same as in [3] but UniprotKB_E.coli_K-12 database (updated 29.04.2019) was used. From intensities of identified peptides L/M ratios (L – light, unlabeled; M – medium-heavy, standard) were calculated for each protein. L/M ratios were normalized against the average L/M ratio of all 50S proteins except for bL31.

Analysis of the relative viability of *E. coli* strains by fluorescence-activated cell sorter

To assess the viability of bacterial cells MG, the A, the B and ΔAB strains in *ΔlacIΔlacZ* background were cultivated separately in M9 medium supplemented with 0.4% glucose at 25°C or 37°C. First, overnight cultures were diluted to start A_{600} 0.1 (final volume 20 ml) and grown at the specified temperature in a shaker for 6 days. Then, the optical density of cultures was measured and dilutions to A_{600} 0.02 were prepared. Next, combined reagent mixture was prepared by adding 3 µl SYTO 9 and 3 µl propidium iodide (both from LIVE/DEAD BacLight Bacterial Viability Kit) to 100 µl M9 medium. 5 µl of this mixture was added to the 100 µl of the cell culture dilution and incubated in the shaker for 30 minutes at 30°C in dark. Then, samples were analyzed with fluorescence-activated cell sorter FACS Aria II (BD Biosciences1, Becton Drive, Franklin Lakes, NJ). Both fluorescent dyes were excited with a 488nm laser with nominal power of 20mW. SYTO 9 was detected using 502LP and 530/30 optical filters and PI was detected using 610LP and 616/23 optical filters. BD FACSFlow was used as sheath fluid in all experiments. Data was analyzed using BD FACSDiva software ver. 6.1.3. As a result of these measurements the number of dead cells and live cells were obtained for each sample. The fraction of live cells was calculated as the number of live cells divided by the sum of dead and live cells. The experiment was conducted in at least six biological replicates.

Analysis of 70 S formation *in vitro*.

70S ribosomes and 50S subunits were purified essentially as described in (2). For *in vitro* reassociation of 70S 5 units (A260) of wild-type 30 subunits (purified from MRE600 strain) with 5 units (A260) of 50S subunits from wild-type or A- and B-strains. Reassociation reactions were carried out for 30 min at 37 °C in 100 µl OV buffer. For each mutant, parallel assays with three different Mg^{2+} concentrations (OV buffer with 6, 8, and 10 mM) were performed. Samples were diluted to 500µl with respective OV buffer, loaded onto a 10–25% sucrose gradient in OV buffer with the same magnesium concentration used for the reassociation reaction and centrifuged at 50,000g for 17 h using a SW-28 rotor (Beckman).

References

1. Neidhardt, F. C., Bloch, P. L. & Smith, D. F. Culture Medium for Enterobacteria. *J. Bacteriol.* **119**, 736–747 (1974).
2. Lilleorg, S., Reier, K., Remme, J. & Liiv, A. The Intersubunit Bridge B1b of the Bacterial Ribosome Facilitates Initiation of Protein Synthesis and Maintenance of Translational Fidelity. *Journal of Molecular Biology* **429**, 1067–1080 (2017).
3. Lilleorg, S. *et al.* Bacterial ribosome heterogeneity: Changes in ribosomal protein composition during transition into stationary growth phase. *Biochimie* **156**, 169–180 (2019).
